# Molecular Modulation of the Crosstalk Between TDP-43 and SOD1

**DOI:** 10.3390/ijms27083409

**Published:** 2026-04-10

**Authors:** Gabriela D. Ribeiro, Daniela D. Queiroz, José R. Monteiro-Neto, Ellen Gerhardt, Gabriel F. de Souza, Paola C. S. C. Albino, Luan H. Paranhos, Tiago F. Outeiro, Elis C. A. Eleutherio

**Affiliations:** 1 Institute of Chemistry, Federal University of Rio de Janeiro (UFRJ), Av. Athos da Silveira Ramos, 149, Rio de Janeiro 21941-909, RJ, Brazil; gdelaqua@gmail.com (G.D.R.); dani_dias4@yahoo.com.br (D.D.Q.); jraphamonteiro0810@gmail.com (J.R.M.-N.); gabrielftts2000@gmail.com (G.F.d.S.); pcristina@eq.ufrj.br (P.C.S.C.A.); luan.holanda@outlook.com (L.H.P.); 2 Department of Experimental Neurodegeneration, Center for Biostructural Imaging of Neurodegeneration, University Medical Center Göttingen, 37073 Göttingen, Germany; egerhar1@gwdg.de (E.G.); touteiro@gmail.com (T.F.O.); 3 Max Planck Institute for Multidisciplinary Sciences, 37075 Göttingen, Germany; 4 Translational and Clinical Research Institute, Faculty of Medical Sciences, Newcastle University, Framlington Place, Newcastle Upon Tyne NE2 4HH, UK; 5 Deutsches Zentrum für Neurodegenerative Erkrankungen (DZNE), 53127 Göttingen, Germany

**Keywords:** TDP-43, SOD1, amyotrophic lateral sclerosis, glycation, proteinopathy

## Abstract

Glycation of superoxide dismutase 1 (SOD1) has been shown to modulate the cytosolic levels of phosphorylated TAR DNA-binding protein 43 (TDP-43), a hallmark of amyotrophic lateral sclerosis (ALS) pathology. In this study, we investigated the interaction between TDP-43 and SOD1 and assessed how methylglyoxal (MGO)-induced glycation and the ALS-associated G93A SOD1 mutation affect this interplay in H4 cells. MGO exposure reduced SOD1 activity and TDP-43 phosphorylation in cells expressing WT SOD1, but not in those expressing G93A SOD1. Both WT and mutant SOD1 interacted with TDP-43 in the nucleus and cytosol; however, cytosolic interactions were more prevalent in G93A-expressing cells. Although MGO did not significantly alter the overall interaction between TDP-43 and WT SOD1, it induced cytosolic inclusion formation at 0.4 mM, a concentration associated with reduced cell viability. These inclusions did not colocalize with stress granules, indicating alternative aggregation pathways. Treatment with cyclosporin A, which inhibits the phosphatase calcineurin, decreased both TDP-43–WT SOD1 inclusions and cytosolic interactions between TDP-43 and G93A SOD1. Together, these findings suggest that SOD1 damage, induced by glycation or ALS-linked mutation, may affect TDP-43 phosphorylation status and promote its cytosolic mislocalization and aggregation, providing new insights into ALS-associated proteinopathy.

## 1. Introduction

Amyotrophic lateral sclerosis (ALS) is a rare neurodegenerative disease that affects upper and lower motor neurons, leading to weakness of voluntary muscles and respiratory dysfunction. With rapid progression, patients have a life expectancy of about three years after symptom onset, with most cases resulting in respiratory failure [1]. ALS can be classified as familial (5–10%) or sporadic (90–95%), and proteinopathy is a pathological hallmark, with 97% of cases presenting TAR DNA-binding protein 43 (TDP-43) loss of function and/or aggregation [2]. TDP-43 contains significant intrinsically disordered regions, particularly within its C-terminal domain, which play a crucial role in pathological aggregation associated with ALS [3]. Pathological heterogeneity has been observed in cases carrying mutant Cu-Zn superoxide dismutase (SOD1), where TDP-43 proteinopathy is not always present, and cytoplasmic protein inclusions exhibit a different composition [4].

TDP-43 is an RNA-binding protein involved in gene expression regulation [5]. It is present mainly in the nucleus, where it participates in pre-mRNA splicing, transcriptional repression, and autoregulation of its gene [6]. TDP-43 can also be shuttled to the cytoplasm, where it plays an important role on mRNA stability, transport, and in the formation and regulation of stress granules [5,6]. In addition to aggregation, mislocalization and hyperphosphorylation of TDP-43 are also thought to contribute to ALS progression [7].

Although twelve phosphorylation sites in the C-terminal domain of TDP-43 have been associated with ALS, phosphorylation at S369, S379, S403/404, and S409/410 are more consistently reported [8,9]. Several studies describe TDP-43 phosphorylation as a regulatory event, controlled by kinases such as casein kinases 1 and 2 (CK1/2) [10,11], tubulin kinases 1 and 2 (TTBK1/2) [12] and cell division cycle 7 (CDC7) [13]. TDP-43 phosphorylation influences splicing regulation, cellular localization [9], condensate formation, and solubility [8]. On the other hand, calcineurin dephosphorylates and colocalizes with TDP-43 in human brain. This phosphatase is responsible for dephosphorylating serine sites in the C-terminal domain of the protein [14]. SOD1 physically interacts with calcineurin, enhancing its phosphatase activity [15] and protecting against its oxidative inactivation [16]. SOD1 seems also been involved with the stability of a variety of eukaryotic CK1 [17]. Taken together, these results indicate that SOD1 might influence TDP-43 phosphorylation.

SOD1 is an antioxidant enzyme present in all human tissues, involved in superoxide dismutation mainly in the mitochondrial intermembrane space, maintaining redox homeostasis alongside other enzymes [18]. SOD1 plays other roles, such as regulating the response against oxidative stress in the nucleus, and the shift from respiration to aerobic glycolysis. Over 200 mutations have been described in SOD1 in ALS [19], with G93A being the most studied due to its association with decreased enzymatic activity, conformational instability, increased oxidative stress, and aggregation propensity [20,21].

Besides genetic mutations, post-translational modifications (PTMs) play a critical role in altering the conformation, stability, and function of SOD1. These modifications are highly relevant to the protein’s normal physiological function and its potential role in diseases. Molecular damage in SOD1, such as glycation by methylglyoxal (MGO) at Lys122 and Lys128 can induce structural instability and promote aggregation [22,23,24]. MGO is a highly reactive byproduct of glycolysis that has been associated with neurodegenerative diseases including ALS [25,26].

Previously, SOD1 and TDP-43 proteinopathies were considered mutually exclusive in ALS. However, some studies have reported both TDP-43 mislocalization to the cytoplasm and increased phosphorylation in a G93A SOD1 mouse model, and in one patient carrying the G85S SOD1 mutation [27,28]. Furthermore, co-deposition of SOD1 and phosphoTDP-43 have been observed in both familial and sporadic cases of ALS [29], suggesting a potential synergistic interaction between these two proteinopathies in the disease’s progression.

Here, we examined the effects of glycation and G93A mutation on SOD1 activity and on the C-terminal phosphorylation of TDP-43 in H4 cells, a widely used model for investigating complex neurological disorders, like ALS [20,21,30]. H4 cells are derived from human brain tissue, which allows for more relevant studies on human-specific pathology. Using the bimolecular fluorescence complementation (BiFC) assay, which allows to observe protein interactions in vivo, we also assessed whether TDP-43 interacts with SOD1 (WT or mutant G93A) and how exposure to MGO affects these interactions. We hypothesized that structural damage to SOD1, either by ALS-associated mutation or glycation by MGO, could differentially modulate its interaction with TDP-43 and influence TDP-43 phosphorylation and localization, thereby promoting cytosolic accumulation and aggregation associated with ALS pathology.

## 2. Results

### 2.1. Methylglyoxal Induces Dose-Dependent Cytotoxicity in H4 Cells

While the normal concentration of methylglyoxal (MGO) in human blood plasma typically ranges from 100 to 120 nM, certain pathological conditions, such as neurodegenerative disorders, can cause a significant increase in these levels. A previous study found that MGO levels in the cerebrospinal fluid of patients with Alzheimer’s disease can reach 0.06 mM [31].

**Figure 1:**
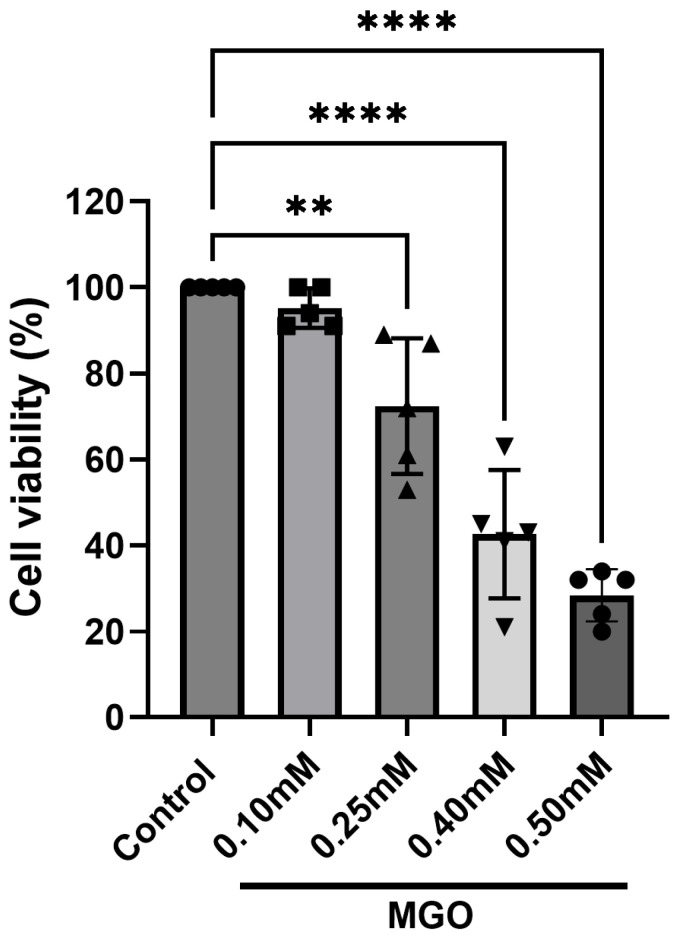
**Cellular viability after MGO treatment.** H4 cells were treated with four different MGO concentrations for 24 h. The cellular viability was determined by MTT assay, as described in Section 4, and the results were expressed as Mean ± SD of the percentage of viable cells. Five independent experiments were performed (n = 5 independent cell culture preparations), and One-Way ANOVA with Dunnett’s multiple comparison test was used for statistical analysis with significance level of ** *p* < 0.01 and **** *p* < 0.0001.

To establish a cellular model of glycation stress, we first evaluated the toxicity of MGO in H4 cells using an MTT viability assay. Exposure to increasing MGO concentrations resulted in a clear dose-dependent reduction in cell viability (Figure 1). Cells treated with 0.1 mM and 0.25 mM MGO retained high viability (~100% and ~80%, respectively), whereas 0.5 mM MGO caused severe cytotoxicity, reducing viability to ~30%. Based on these results, a concentration of 0.4 mM MGO—corresponding to approximately 50% cell viability—was selected for subsequent experiments to model substantial but sublethal glycation stress. During the development of age-related neurodegenerative diseases, brain tissues are exposed to progressively increasing levels of MGO, subjecting neuronal cells to prolonged exposure to elevated MGO concentrations that can ultimately lead to cellular damage and death. To simulate this physiological process, H4 cells were exposed to high levels of MGO, and we selected the concentration that produced a significant reduction in cell viability.

### 2.2. SOD1 Mutation and Glycation Stress Differentially Impair SOD1 Activity and TDP-43 Phosphorylation

Previous studies have demonstrated that MGO modifies WT SOD1, confirming its toxic and aggregation-promoting properties, in agreement with findings reported for other neurodegenerative disorders [25,32,33]. SOD1 is involved in several molecular processes that are essential for maintaining neuronal integrity and function; therefore, its dysfunction, caused by damage or mutations, may have detrimental effects on neuronal health [21].

**Figure 2:**
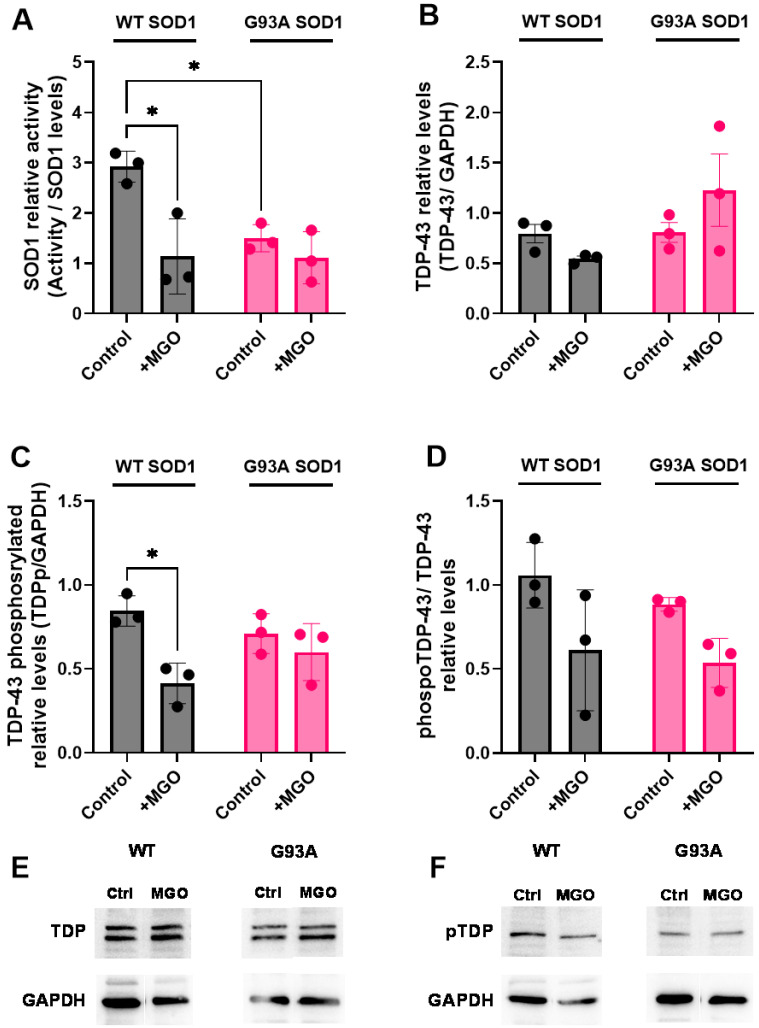
**Effects of MGO-induced stress on SOD1 activity and TDP-43 levels.** Cells transfected with WT or G93A SOD1 plasmids were treated with 0.4 mM MGO for 24 h. (**A**) SOD1 activity was determined by non-denaturing gel zymography using nitroblue tetrazolium (NBT) staining, as described in Section 4, and normalized to SOD1 protein levels. (**B**) Total TDP-43 and (**C**) phosphorylated TDP-43 (Ser409) levels were determined by immunoblotting and normalized to GAPDH. (**D**) Ratio of phosphorylated TDP-43 to total TDP-43 normalized levels. Data are presented as Mean ± SD from at least three independent experiments (n = 3 independent cell culture preparations). Statistical analyses for (**A**,**B**) were performed using two-way ANOVA followed by Tukey’s multiple comparisons test. For (**C**,**D**), comparisons between SOD1 WT and G93A should be interpreted with caution due to the absence of same-gel analysis. In this case, statistical significance between control and MGO groups was assessed using a two-tailed *t*-test. Statistical significance was defined as * *p* < 0.05. (**E**,**F**) show representative images of total and phospho (Ser 409)-TDP-43 blots, respectively. The native gel images showing SOD1 activity and the original blots presenting SOD1, TDP-43 and phospho-TDP-43 levels are shown in Supplementary Material (Figures S1 and S2).

Then, we next examined how MGO stress and ALS-associated SOD1 mutation affect SOD1 enzymatic function. H4 cells were transfected with plasmids encoding WT or G93A SOD1, and SOD1 relative activity was assessed. Cells expressing G93A SOD1 displayed significantly reduced SOD1 activity compared with WT-expressing cells, even in the presence of endogenous SOD1 (Figure 2A), consistent with the known loss-of-function associated with this mutation [34]. MGO treatment markedly reduced WT SOD1 activity, in agreement with previous reports showing that glycation compromises SOD1 function [24]. Notably, MGO did not further reduce the activity of G93A SOD1, suggesting that the mutant enzyme is already maximally impaired.

Because SOD1 has been implicated in regulating TDP-43 phosphorylation through stabilization of both casein kinase 1 (CK1/2) and calcineurin [15,17], we next analyzed total and phosphorylated TDP-43 levels. Immunoblot analyses revealed no significant differences in total TDP-43 or phospho-Ser409 TDP-43 levels between WT and G93A SOD1-expressing cells under basal conditions (Figure 2B,C). However, MGO treatment selectively reduced TDP-43 phosphorylation in WT SOD1-expressing cells, while no significant change was observed in cells expressing G93A SOD1 (Figure 2C,D). The levels of TDP-43 and phospho-TDP-43 obtained from samples of cells expressing SOD1 WT or G93A were analyzed on separate gels; therefore, direct quantitative comparisons between WT and mutant variants are not reliable due to potential inter-gel variability, and interpretations are limited to comparisons within each condition (control X MGO). Our results suggest that glycation-induced loss of WT SOD1 activity disrupts phosphorylation-dependent regulation of TDP-43, whereas this regulatory axis is already compromised in the mutant context. The exact role of TDP-43 phosphorylation is not known and the phospho-form can be found in the nucleus and in the cytosol (Supplementary Material—Figure S3).

### 2.3. TDP-43 Interacts with WT and Mutant SOD1 in Distinct Cellular Compartments

Interactions between TDP-43 and mutant SOD1 have previously been demonstrated by immunoprecipitation [35]; however, an association between TDP-43 and WT SOD1 had not been reported. Despite its widespread use, immunoprecipitation presents several limitations for studying protein–protein interactions, including dependence on high-quality antibodies, limited sensitivity for weak interactions, background noise from non-specific binding, the potential for false negatives due to antibody-mediated interference, and difficulty in distinguishing direct from indirect interactions.

Bimolecular Fluorescence Complementation (BiFC) overcomes many of these limitations by enabling direct, real-time visualization of protein interactions in living cells. This approach allows precise determination of subcellular localization and offers high sensitivity without the need for additional reagents. Because BiFC is performed in living cells, it preserves native cellular conditions and post-translational modifications, thereby providing more physiologically relevant results. In this assay, fluorescence is reconstituted only when two proteins interact, each being fused to a non-fluorescent, truncated fragment of a fluorescent protein (e.g., Venus). Consequently, BiFC enables the detection of interactions of two proteins, which may further assemble into higher-order oligomeric structures.

**Figure 3:**
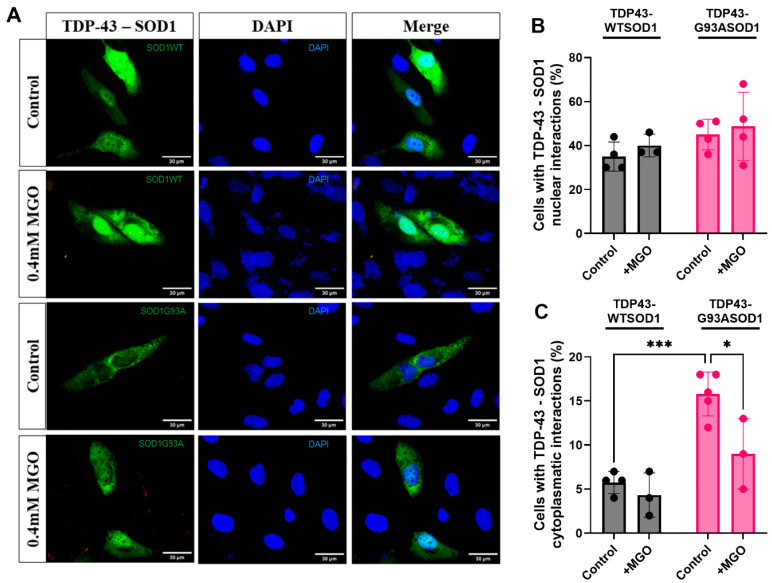
**Subcellular distribution of TDP-43–SOD1 interactions.** (**A**) Representative images of H4 cells expressing the BiFC system (VN-TDP-43 and WT—lanes 1 and 2—or mutant—lanes 3 and 4 -SOD1–VC plasmids) after 24 h of MGO treatment or under control conditions. Cells were analyzed by fluorescence microscopy, and fluorescent cells were quantified. Scale bar, 30 μm; magnification, ×1000. At least 100 cells were counted per condition. BiFC fluorescence intensity was quantified using ImageJ, version 1.52a. The percentages of cells exhibiting TDP-43–SOD1 interactions confined to the (**B**) nucleus (stained with DAPI) or (**C**) cytosol are shown. Data are presented as Mean ± SD from at least three independent experiments (n = 3 independent cell culture preparations). Statistical analysis was performed using two-way ANOVA followed by Tukey’s multiple comparison test; * *p* < 0.05, *** *p* < 0.001.

To assess whether SOD1 physically interacts with TDP-43 in living cells and to determine the subcellular localization of this interaction, H4 cells were transfected with Venus-tagged TDP-43 and either WT or G93A SOD1 constructs. Reconstitution of Venus fluorescence confirmed that TDP-43 interacts with both WT and mutant SOD1 (Figure 3A). This interaction was robust, as more than 50% of cells exhibited fluorescence under all tested conditions. As negative controls, cells transfected solely with VN–TDP-43 and VC–SOD1 plasmids showed no detectable fluorescence. Similarly, transfection with the reciprocal VC–TDP-43 and VN–SOD1 constructs yielded no differences in fluorescence, confirming the specificity of the observed interactions.

Quantitative analysis revealed that TDP-43–SOD1 interactions occurred predominantly in the nucleus, with approximately 40% of cells displaying nuclear BiFC signal (Figure 3B). However, cells expressing G93A SOD1 exhibited a markedly higher proportion of cytosolic TDP-43–SOD1 interactions—nearly threefold higher than WT SOD1-expressing cells (Figure 3C). This shift likely reflects the impaired nuclear localization and altered trafficking of mutant SOD1 reported previously [20]. Importantly, MGO treatment did not significantly alter the distribution of TDP-43–WT SOD1 interactions but reduced the cytosolic TDP-43–G93A SOD1 interaction to levels comparable to WT SOD1, suggesting that glycation stress modulates mutant-specific interaction dynamics.

### 2.4. Calcineurin Inhibition Shifts TDP-43–WT SOD1 Interaction Toward the Nucleus

SOD1 stabilizes calcineurin, the phosphatase that dephosphorylates TDP-43 at Ser409/410 [15]. To investigate how TDP-43 phosphorylation influences the subcellular compartment in which the TDP-43–SOD1 interaction occurs, cells were treated with the calcineurin inhibitor cyclosporin A (CsA). Previous studies have shown that inhibition of calcineurin in HEK293 cells leads to the accumulation of phosphorylated TDP-43 at Ser409/410 [14].

**Figure 4:**
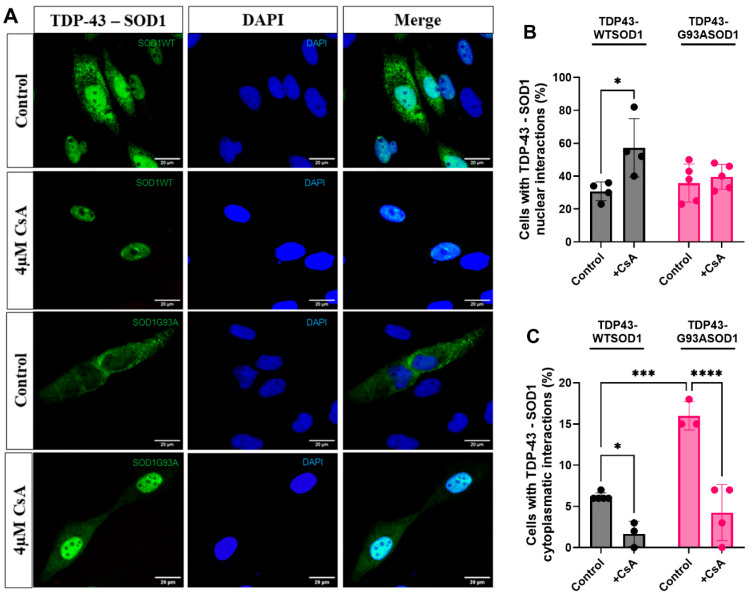
**Subcellular distribution of TDP-43–SOD1 interactions after calcineurin inhibition by CsA.** (**A**) Representative pictures of H4 cells expressing VN-TDP-43 and WT (lanes 1 and 2) or mutant (lanes 3 and 4) SOD1–VC following 24 h treatment with 4 µM CsA or under control conditions. Cells were analyzed by fluorescence microscopy, and fluorescent cells were quantified. Scale bar, 20 μm; magnification, ×1000. At least 100 cells were counted per condition. Fluorescence intensity was quantified using ImageJ. (**B**,**C**) show the percentages of cells showing TDP-43–SOD1 interactions confined to the nucleus (stained with DAPI) or cytosol, respectively. Data are presented as Mean ± SD from at least three independent experiments (n = 3 independent cell culture preparations). Statistical analysis was performed using two-way ANOVA followed by Tukey’s multiple comparison test; * *p* < 0.05, *** *p* < 0.001, **** *p* < 0.0001.

Our results indicate that inhibition of calcineurin favors the nuclear interaction between TDP-43 and WT SOD1 (Figure 4A,B). In contrast, no significant effect was observed on the nuclear interaction between TDP-43 and G93A SOD1. Moreover, calcineurin inhibition reduced the cytosolic interaction between TDP-43 and both WT and mutant SOD1 (Figure 4C), with a more pronounced reduction observed for the mutant, as the proportion of cells exhibiting cytosolic TDP-43–WT SOD1 interactions was already very low.

These findings suggest that increased phosphorylation of TDP-43 may weaken or destabilize TDP-43–SOD1 interactions, particularly in the cytoplasm. These results support a model in which active calcineurin promotes cytosolic TDP-43–SOD1 association, at least in the WT SOD1 context. Notably, mutant SOD1 displays impaired nuclear localization [20], which may explain why calcineurin inhibition did not alter the nuclear interaction between TDP-43 and G93A SOD1.

### 2.5. Glycation Stress Promotes Cytosolic TDP-43–SOD1 Inclusions Independent of Stress Granules

**Figure 5:**
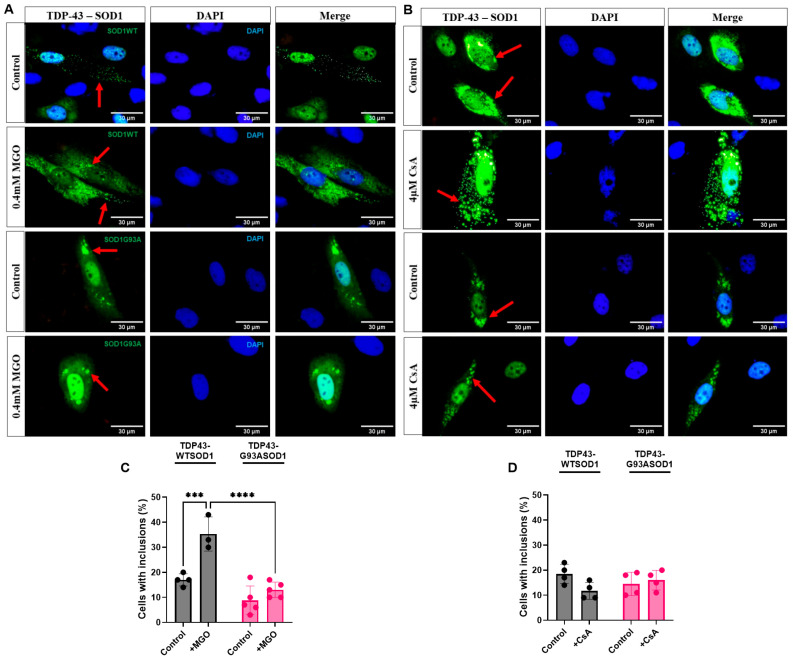
**MGO increases the percentage of cells containing TDP-43–WT SOD1 inclusions.** Representative images of cells containing TDP-43–WT SOD1 (lanes 1 and 2) or TDP-43–G93A SOD1 (lanes 3 and 4) inclusions after 24 h exposure to 0.4 mM MGO (**A**) or 4 µM CsA (**B**), a calcineurin inhibitor. Cells were analyzed by fluorescence microscopy, and fluorescent cells displaying inclusions (indicated by red arrows) were quantified. Nuclei were stained with DAPI. Scale bar, 30 μm; magnification, ×1000. At least 100 cells were counted per condition (24 h at control condition, MGO stress, or CsA treatment). Fluorescence intensity was quantified using ImageJ. (**C**,**D**) show the percentage of cells containing TDP-43–SOD1 inclusions following MGO-induced stress or CsA treatment, respectively. Data are presented as Mean ± SD from at least three independent experiments (n = 3 independent cell culture preparations). Statistical analysis was performed using two-way ANOVA followed by Tukey’s multiple comparison test; *** *p* < 0.001, **** *p* < 0.0001.

Microscopic analysis revealed the presence of cytosolic inclusions containing both TDP-43 and SOD1 even under basal conditions (Figure 5A,B). Exposure to MGO significantly increased the proportion of cells displaying these inclusions. In cells expressing WT SOD1, MGO induced a twofold increase in cytosolic TDP-43–SOD1 inclusions (Figure 5C). MGO stress did not increase inclusions in G93A SOD1-expressing cells, likely reflecting the intrinsic aggregation propensity of the mutant protein [20]. Contrary to MGO, inhibition of calcineurin by CsA did not affect the percentage of cells with TDP-43-SOD1 inclusions. The dominant effect of CsA appeared to be on the localization of TDP-43–SOD1 interactions rather than inclusion formation per se.

**Figure 6:**
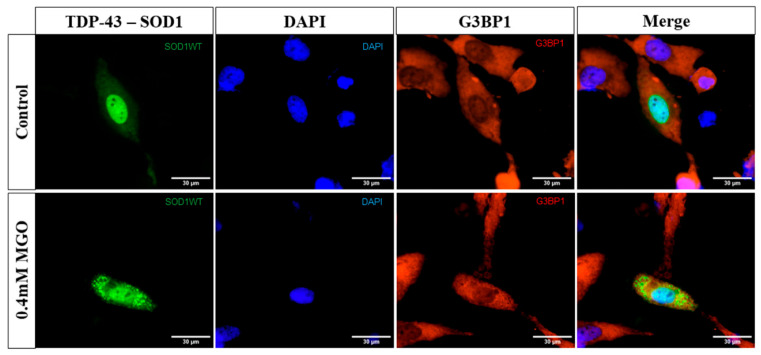
**TDP-43–WT SOD1 inclusions do not colocalize with stress granules.** Representative images of H4 cells expressing TDP-43–WT SOD1, exposed or not to 0.4 mM MGO-induced stress. G3BP1 (red) was used as a marker of stress granule (SG) formation, and cell nuclei were stained with DAPI (blue). Cells were analyzed by fluorescence microscopy, and inclusions were visualized by BiFC-tagged TDP-43 and SOD1 proteins (green). At least 50 cells per condition were analyzed from three independent experiments (n = 3 independent cell culture preparations). Scale bar, 30 μm; magnification, ×1000.

Because both TDP-43 and SOD1 have been reported to localize to stress granules (SGs) [24,36], we assessed whether the observed inclusions corresponded to SGs by co-staining for the SG marker G3BP1. Under MGO stress, G3BP1 displayed a diffuse staining pattern, indicating an absence of SG formation. Accordingly, TDP-43–SOD1 inclusions did not colocalize with SGs (Figure 6), suggesting that these structures represent distinct, stress-induced cytosolic assemblies rather than canonical SGs.

## 3. Discussion

In this study, we investigated how MGO exposure and the ALS-associated G93A mutation, both of which may impair SOD1 functionality, affect SOD1–TDP-43 interactions, their subcellular localization, and the levels of phosphorylated TDP-43, a form commonly found in cytosolic aggregates in most ALS cases. Because of its high reactivity, MGO detoxification relies on glyoxalase enzymes that convert MGO into D-lactate in a glutathione (GSH)-dependent reaction. A decline in glyoxalase 1 activity has been reported in elderly individuals [37], and analyses of the motor cortex in ALS patients have revealed reduced GSH levels [38]. These impairments could alter MGO clearance and exacerbate oxidative stress, suggesting that genetic predispositions or lifestyle factors, though negligible in young individuals, may become risk factors for ALS with aging. ALS remains an age-associated neurodegenerative disease of largely unknown etiology.

Using the BiFC system, we confirmed that TDP-43 can physically interact with both WT and mutant G93A SOD1 in living cells. We further observed that SOD1 predominantly binds to TDP-43 in the nucleus. Interestingly, inhibition of calcineurin promoted nuclear localization of TDP-43-SOD1. Studies employing C-terminal phosphomimetic TDP-43 variants indicate that phosphorylation does not impair nuclear import or RNA regulatory functions [39]. Thus, active calcineurin likely facilitates the cytosolic localization of TDP-43 and SOD1. Debates persist regarding the function of TDP-43 phosphorylation; current evidence suggests it plays a regulatory role in localization and solubility, potentially preventing pathological condensation [8,9].

In the cytoplasm, TDP-43 may undergo CK1-dependent phosphorylation, which requires active SOD1 and SOD1–CK1 interaction [17]. Hence, we hypothesize that SOD1 could serve as a molecular bridge between TDP-43 and CK1, facilitating TDP-43 phosphorylation. However, this hypothesis goes beyond the data obtained in the present study and requires further experimental validation. Given that SOD1 also modulates phosphatase activity, it is plausible that CK1 activation antagonizes calcineurin function. The specific cellular signals that switch SOD1 activity from supporting calcineurin to activating CK1 remain unknown but may be stress dependent.

Our data indicate that phosphorylation may promote TDP-43 solubility. Phosphorylation of serine residues can alter the charge properties of TDP-43, potentially enhancing its solubility and shifting condensates toward a more dynamic and less aggregated state. Previous studies have suggested that these changes may facilitate the recruitment of TDP-43 to stress granules (SGs) under certain stress conditions [39]. However, in our experimental conditions, MGO treatment did not induce SG formation. Whether TDP-43 reaches SGs in complex with SOD1 therefore remains unclear, although both proteins have been reported to localize to SGs under other stress conditions.

Prolonged or severe stress can inactivate SOD1, leading to CK1 inactivation and reduced TDP-43 phosphorylation. Consistent with this, we observed reduced levels of phospho-TDP-43 and increased cytosolic TDP-43–SOD1 inclusions under an acute MGO stress, without significant changes in their overall distribution. This finding indicates that glycation-induced SOD1 damage may affect downstream protein homeostasis rather than directly increasing the cytosolic interaction between these proteins. Instead, MGO-mediated structural alterations may favor the formation of cytosolic inclusions containing SOD1 and TDP-43. Conversely, CsA treatment modestly reduced these inclusions, consistent with the absence of stress during CsA treatment. Intense stress conditions favor the accumulation of misfolded proteins, overwhelming SG capacity and resulting in cytoplasmic TDP-43 accumulation and aggregation [36]. This phenomenon may explain the presence of phosphorylated TDP-43 inclusions in advanced ALS stages.

In cells expressing G93A SOD1, we observed a greater cytosolic interaction with TDP-43, potentially reflecting the mutant’s impaired nuclear localization [20,30]. The G93A SOD1 failure to translocate to the nucleus may account for the increased interaction of the mutant SOD1 with TDP-43 in the cytosol, as compared to the WT form. Nuclear SOD1 has essential transcriptional roles, regulating genes involved in oxidative stress responses and protecting DNA from oxidative damage [40]. Therefore, reduced activity and nuclear localization of G93A SOD1 may increase cellular vulnerability to oxidative stress and genotoxicity. Clinically, patients with familial ALS linked to SOD1 mutations exhibit SOD1-positive, but not TDP-43-positive, inclusions and often show earlier symptom onset than sporadic ALS patients. In our BiFC assays, the TDP-43–SOD1 interaction produced fluorescence without revealing SOD1-only aggregates, supporting the notion that mutant SOD1 primarily drives TDP-43 mislocalization rather than independent aggregation.

**Figure 7:**
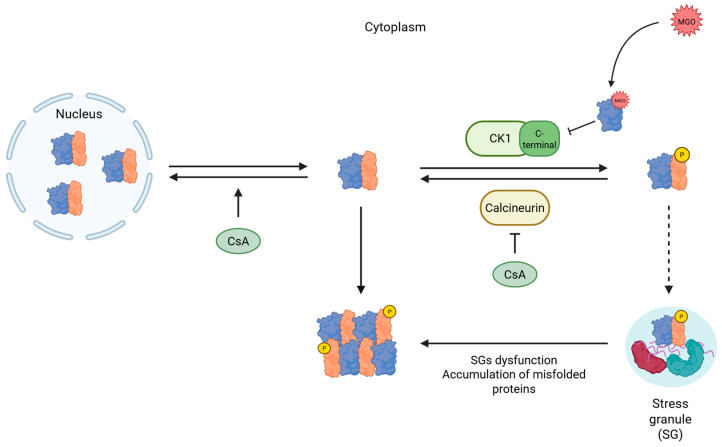
**Proposed mechanism for the effect of MGO on SOD1-TDP-43 interaction.** SOD1 (blue) interacts with TDP-43 mainly in the nucleus. The level of phosphoTDP-43 (orange) appears to depend on SOD1, which influences both calcineurin and CK1. In response to MGO, SOD1 activity decreases, reducing phospho-TDP-43, which promotes inclusion formation. Phosphorylation of TDP-43 increases its solubility, reducing TDP-43 proteinopathy, a hallmark of ALS. The localization of TDP-43 in stress granules (SGs) also protects against proteinopathy. However, when the capacity of SGs to store TDP-43 is exceeded, potentially due to the accumulation of unfolded proteins within SGs, TDP-43 aggregates in the cytosol.

Based on our findings, we propose a model in which MGO perturbs TDP-43–SOD1 dynamics, thereby influencing TDP-43 localization and inclusion formation (Figure 7). Overall, our study underscores the pivotal role of SOD1 in maintaining TDP-43 homeostasis. This conclusion is consistent with previous findings [26], which detected misfolded SOD1 in degenerating regions of both familial and sporadic ALS patients. Furthermore, elevated glycation of the SOD1 electrostatic loop has been reported in all ALS patient spinal cords examined [29], suggesting that the mechanisms described here may contribute broadly to TDP-43 mislocalization in ALS. In addition to clinical observations, abundant evidence from transgenic SOD1 mouse models supports a functional relationship between SOD1 and TDP-43 [27,28], which is in line with the findings presented in this study. We propose that reduced SOD1 activity—whether resulting from MGO-induced modification or ALS-associated genetic mutations—induces cellular dysregulation, leading to TDP-43 aggregation under WT SOD1 conditions or mislocalization in the context of the G93A mutant. Phosphorylation of TDP-43 emerges as a critical determinant of its solubility and nuclear retention, preventing pathological aggregation. Conversely, this phosphorylation-dependent regulation is impaired in G93A SOD1-expressing cells, highlighting the essential role of SOD1 integrity in maintaining TDP-43 homeostasis.

Although our findings provide insight into how MGO-induced SOD1 dysfunction may influence TDP-43 homeostasis, their translational relevance should be interpreted with caution. The concentration of MGO used and the use of H4 cells represent simplified experimental conditions. Future studies using neuronal models and physiologically relevant stress conditions will be necessary to further assess the potential contribution of SOD1 glycation to TDP-43 pathology in ALS.

## 4. Materials and Methods

### 4.1. Cell Culture and MGO Stress

Human neuroglioma cells (H4) we obtained from Prof Tiago F. Outeiro lab (U. Goettingen, Germany). Cells were cultured in Dulbecco’s modified Eagle’s medium (DMEM) (BR30003-05 Nova Biotecnologia, Cotia, Brazil), supplemented with 10% (*v*/*v*) fetal bovine serum (FBS) (10-bio500L—Nova Biotecnologia) and 1% (*v*/*v*) penicillin/streptomycin (BR30110-01—Nova Biotecnologia), at 37 °C in a humidified atmosphere containing 5% CO_2_. H4 cells were challenged with four different MGO concentrations: 0.1 mM, 0.25 mM, 0.4 mM, and 0.5 mM, 24 h after plating. Forty-eight hours after cell plating, the medium with MGO was changed to a DMEM medium (serum-free) with 100 µg/mL MTT (3-(4,5-dimethylthiazol-2-yl)-2,5-diphenyltetrazolium bromide). After two hours, the medium with MTT was disposed of and the cells were incubated in DMSO (dimethyl sulfoxide). Cellular viability was measured using a SpectraMax M2 (Molecular Devices, San Jose, CA, USA) at a wavelength of 570 nm [41].

### 4.2. Cell Transfections

To analyze the effect of MGO on SOD1 activity and phospho-TDP-43 levels, H4 cells (5.0 × 10^5^ cells/mL) were plated in 12-well plates (Kasvi, Haryana, India) in fresh DMEM. The transient transfections were performed using 8 μL of a 2.5 M CaCl_2_ solution, which was added to 62 μL of a plasmid containing the cDNA sequence of human WT SOD1 or the mutant G93A20. Subsequently, 2 × HBS calcium phosphate buffer [50 mM BES (N,N-bis(2-hydroxyethyl)-2-aminoethanesulfonic acid), 280 mM NaCl, 1.5 mM Na_2_HPO_4_·2H_2_O, pH 7.05] was added dropwise to the mixture and thoroughly vortexed. After incubation for 20 min at room temperature, in the dark, the transfection solution was added dropwise to the cultured cells while the plate was gently agitated. Bimolecular Fluorescence Complementation (BiFC) plasmids were employed to investigate the interaction between TDP-43 and SOD1. A larger N-terminal fragment of Venus (VN, corresponding to 1–158 amino acid sequence), and a smaller C-terminal fragment (VC, corresponding to 159–239 amino acid sequence) were used [20,30]. Human TDP-43 cDNA was cloned to the 3′-end of the VN-fragment (VN-TDP-43) and human SOD1 cDNA (WT or G93A) was cloned to upstream of the VC-fragment (SOD1-VC). Transfections were performed by calcium phosphate using equal amounts of plasmids encoding VN-TDP-43 and SOD1-VC (WT or G93A SOD1). Alternatively, cells were transfected with equal amounts of VN–SOD1 and SOD1–VC constructs (WT or G93A) [20] to assess the effect of mutant SOD1 on TDP-43 expression.

### 4.3. Immunoblotting and SOD1 Activity

Forty-eight hours after transfection with WT SOD1 or G93A SOD1 plasmids [20], cells were washed with PBS and then harvested. Cell extracts for enzymatic determinations were obtained by cell incubation in a lysis buffer [NP-40 1% (*v*/*v*), 150 mM NaCl and 50 mM Tris-HCl buffer pH 8.0] containing a protease inhibition mixture (cOmplete, mini, EDTA-free protease mixture inhibitor tablets; Sigma-Aldrich, Saint Louis, MO, USA). Protein concentration was determined using a Bradford assay (B6916—Sigma-Aldrich) using bovine serum albumin (BSA) as a standard. A total of 25 µg of protein extract was separated by 12% SDS–polyacrylamide gel electrophoresis (SDS–PAGE) and transferred to a nitrocellulose membrane. For SOD1 quantification, membranes were blocked overnight in 3% (*w*/*v*) BSA in 1 × PBS-T (50 mM Tris, 150 mM NaCl, 0.05% Tween-20, pH 7.5) and incubated for 1 h at room temperature with primary antibodies: rabbit anti-SOD1 (1:1000; HPA001401, Sigma-Aldrich) and rat anti-α-tubulin (1:10,000; MA180017, Sigma-Aldrich). After washing three times with PBS-T for 10 min, membranes were incubated for 1 h with HRP-conjugated secondary antibodies (1:10,000 anti-rabbit and 1:10,000 anti-rat IgG; Sigma-Aldrich) diluted in 3% BSA/PBS-T. Quantification of TDP-43 and phosphorylated TDP-43 (Ser409) was performed using a well-established methodology. Membranes were incubated for 1 h with rabbit anti-TDP-43 (1:1000; T1705, Sigma-Aldrich) or rabbit anti-phospho-TDP-43 (Ser409) (1:1000; SAB4200223, Sigma-Aldrich), together with mouse anti-GAPDH (1:10,000; AM4300, Thermo Fisher Scientific, Waltham, MA, USA) diluted in 3% BSA/PBS-T. After washing, membranes were incubated for 1 h with HRP-conjugated secondary antibodies (1:10,000 anti-rabbit and 1:10,000 anti-mouse IgG; Sigma-Aldrich). Detection was performed using an ECL Western blotting substrate (Promega, Madison, WI, USA), and SOD1, TDP-43, and phospho-TDP-43 levels were quantified using ImageJ software (version 1.52a). SOD1 activity was measured in situ after native polyacrylamide gel electrophoresis using 25 µg of protein in the presence of riboflavin, N,N,N,N-tetramethylethylenediamine (TEMED) and nitroblue tetrazolium (NBT). Enzyme activity was determined by measuring the ability of superoxide dismutase to inhibit the reduction of NBT by superoxide radicals generated in a riboflavin–TEMED photochemical system. Native gels were digitized using the Fusion Solo 6S system (Vilber Lourmat, Collegien, France) and SOD1 bands were quantified with ImageJ software by measuring the area density of all SOD1 bands [24,30,34]. SOD1 activity was expressed as the ratio between SOD1 enzymatic activity and SOD1 protein levels.

### 4.4. Fluorescence Microscopy

The H4 cells were transfected with VN-TDP-43 and VC-SOD1 plasmids (WT or G93A); and 24 h after, they were stressed with MGO. Forty-eight hours after transfection with VN-TDP-43 and SOD1-VC (WT or G93A mutant), H4 cells were fixed with 4% paraformaldehyde for 30 min at room temperature and washed three times with PBS. For nucleus stain, cells were incubated in DAPI (0.2 µg/mL) for 10 min and washed three times with PBS before the slide preparation. Fluorescence images were captured with an Olympus IX73 fluorescence microscope (Tokyo, Japan), and the fluorescent cells were counted. Quantification was performed exclusively in fluorescent cells, corresponding to cells successfully co-transfected with both BiFC constructs. Cells were classified in two groups: cells with nuclear localization or cytoplasmic localization. Additionally, cells with TDP-43-SOD1 inclusions were counted and classified according to the total number of fluorescent cells. Results reflect the counting of at least 100 fluorescent cells per condition.

### 4.5. TDP-43-SOD1 Colocalization with Stress Granules

H4 cells treated with MGO were submitted to the immunocytochemistry (ICC) protocol 48 h after the transfection with VN-TDP-43 and SOD1-VC plasmids. Cells were washed with PBS and fixed with 4% paraformaldehyde for 30 min at room temperature. After fixation, cells were permeabilized with PBS 0.5% Triton X-100 for 15 min and blocked for 1 h with 3% BSA in PBS-T at room temperature, followed by incubation, overnight at 4 °C, with primary antibody 1:200 anti-G3BP1 (SAB5702394—Sigma-Aldrich), which marks stress granules. Cells were washed three times with PBS-T and incubated with secondary antibody 1:2000 anti-rabbit IgG conjugated with Alexa Fluor 594 (A11012—Invitrogen, ThermoFisher Scientific) for 2 h at room temperature. Nucleus was stained by DAPI, and fluorescence images were captured with an Olympus IX73 fluorescence microscope [23].

### 4.6. Statistical Analysis

Data was analyzed using GraphPad Prism 10.3 software and expressed as mean ± SD of at least 3 independent experiments (independent cell culture preparations). Statistical differences were calculated using One-Way ANOVA or Two-Way ANOVA, as described in the figure subtitle. Significance was expressed: * *p* < 0.05, ** *p* < 0.01, *** *p* < 0.001, **** *p* < 0.0001.

## Data Availability

The original contributions presented in this study are included in the article/Supplementary Material. Further inquiries can be directed to the corresponding author.

## Supplementary Materials

The following supporting information can be downloaded at: https://www.mdpi.com/article/10.3390/ijms27083409/s1.
