# Supplementary material for: Molecular Modulation of the Crosstalk Between TDP-43 and SOD1
Source: Int J Mol Sci. 2026 Apr 10;27(8):3409. doi: 10.3390/ijms27083409 (PMC13116465; doi:10.3390/ijms27083409)
Supplement: Supplementary file 1 [file ijms-27-03409-s001.zip › ijms-4160604-supplementary.pdf]

Supplementary material

A

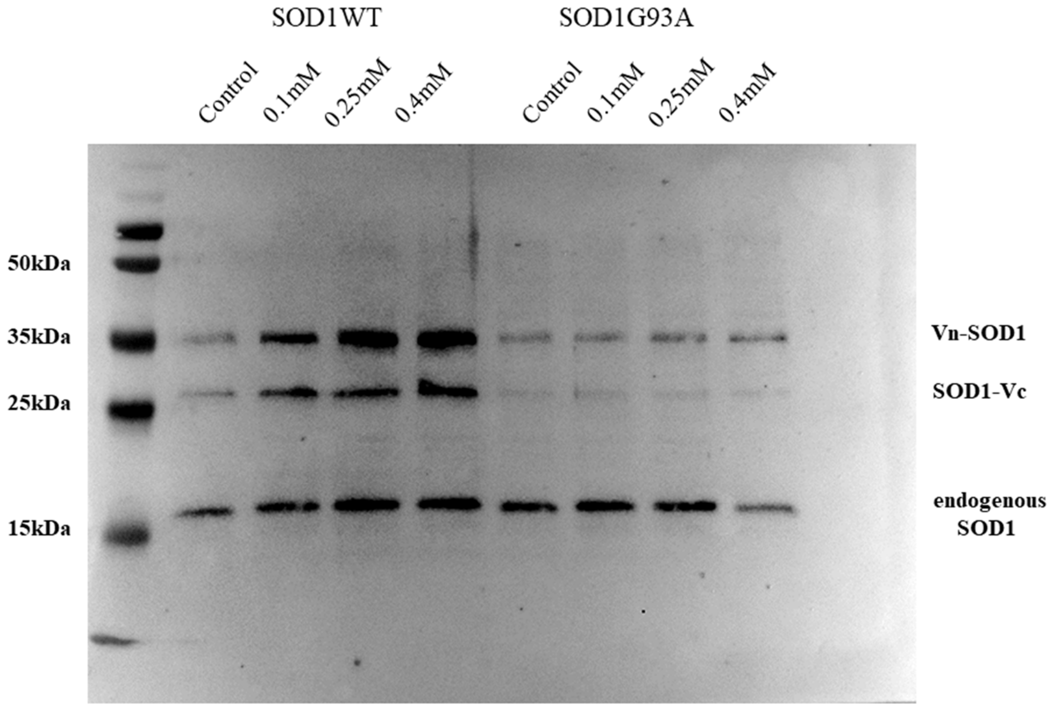

B

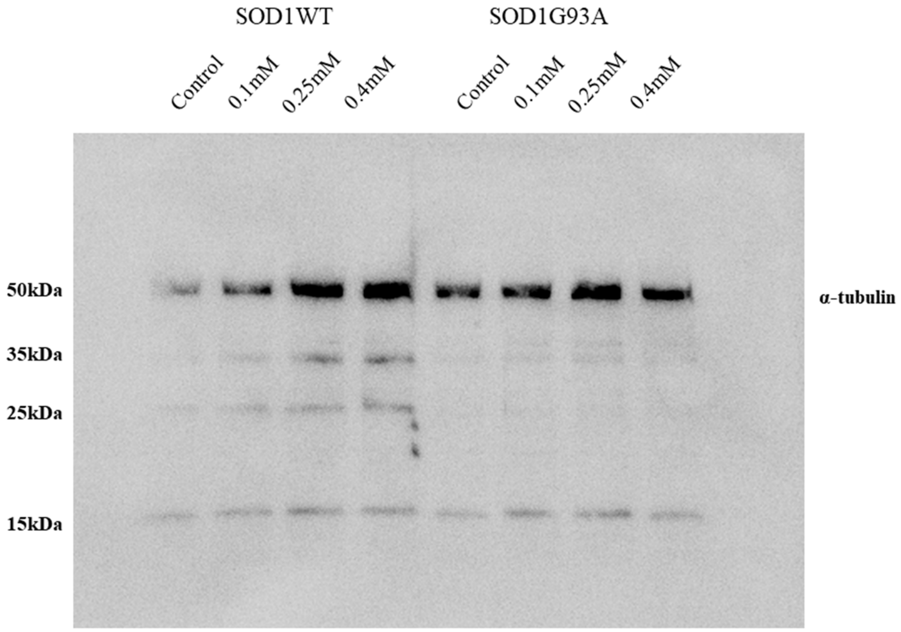

C

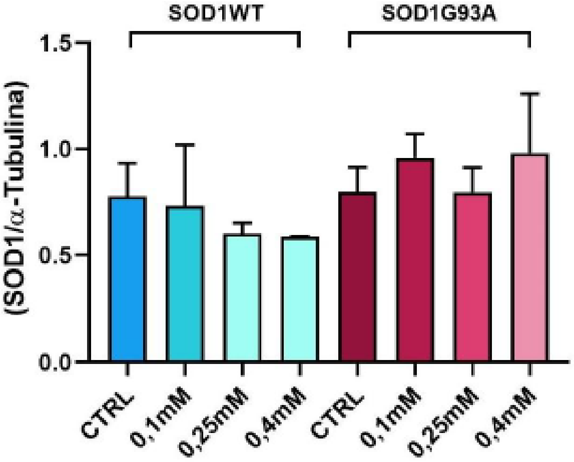
SOD1WT
SOD1G93A
(SOD1/α-Tubulina)
1.5
1.0
0.5
0.0
CTRL
0,1mM
0,25mM
0,4mM
CTRL
0,1mM
0,25mM
0,4mM

D

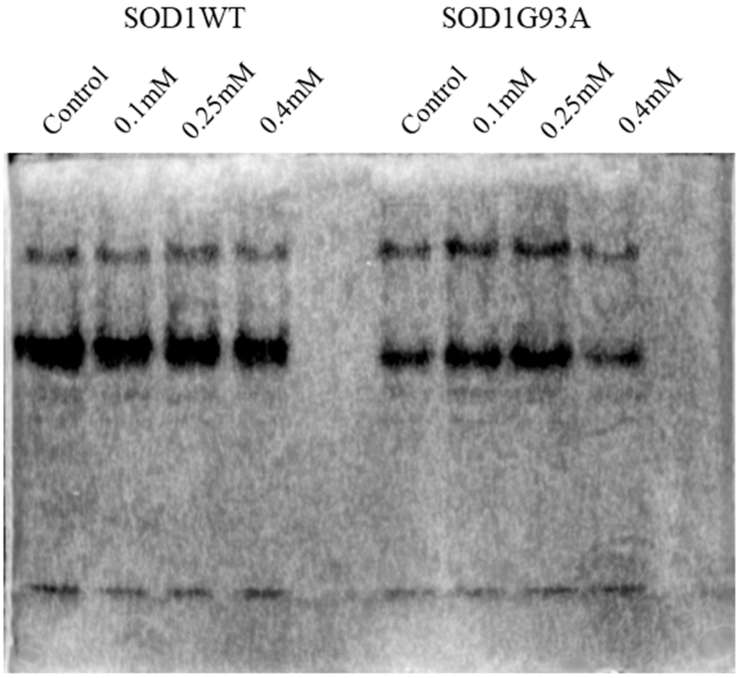
SOD1WT
SOD1G93A
Control
0.1mM
0,25mM
0.4mM
Control
0.1mM
0,25mM
0.4mM

E

Relative SOD1 activity (Activity/SOD1 levels)

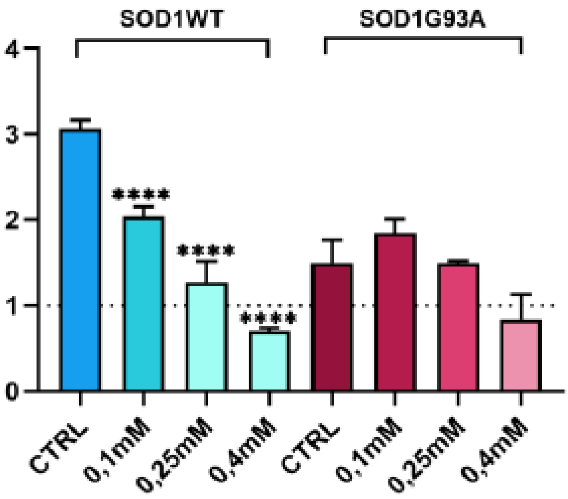

Figure S1: Sod1 levels and activity in cells expressing WT or G93A SOD1 under control condition or after exposure to 0.1, 0.25 or 0.4 mM MGO. Representative image of original blots used to quantify SOD1 (A) and tubulin levels (B). (C) SOD1 levels were normalized to tubulin levels. (D) Representative image of the original native gel (non-denaturing gel) used to determine SOD1 activity. SOD activity was determined based on the areas of the uncolored bands produced due to the inhibition of superoxide radical NBT reduction. The image shows the negative of the native gel which were scanned using the EC3 imaging system, and SOD bands were quantified with ImageJ software by measuring the area density of SOD bands. The bands corresponding to SOD1 activity are those which are greater on the middle of the gel; the upper bands correspond to SOD2 activity and were not used in the quantification. SOD2 is a homotetramer of 100 kDa and PI 7.3 while SOD1 is a homodimer of 32 kDa and PI 5.3. (E) SOD1 activity was normalized to relative SOD1 levels. Statistical analysis was performed using two-way ANOVA; ****$p < 0.05$ (control X MGO in each strain).

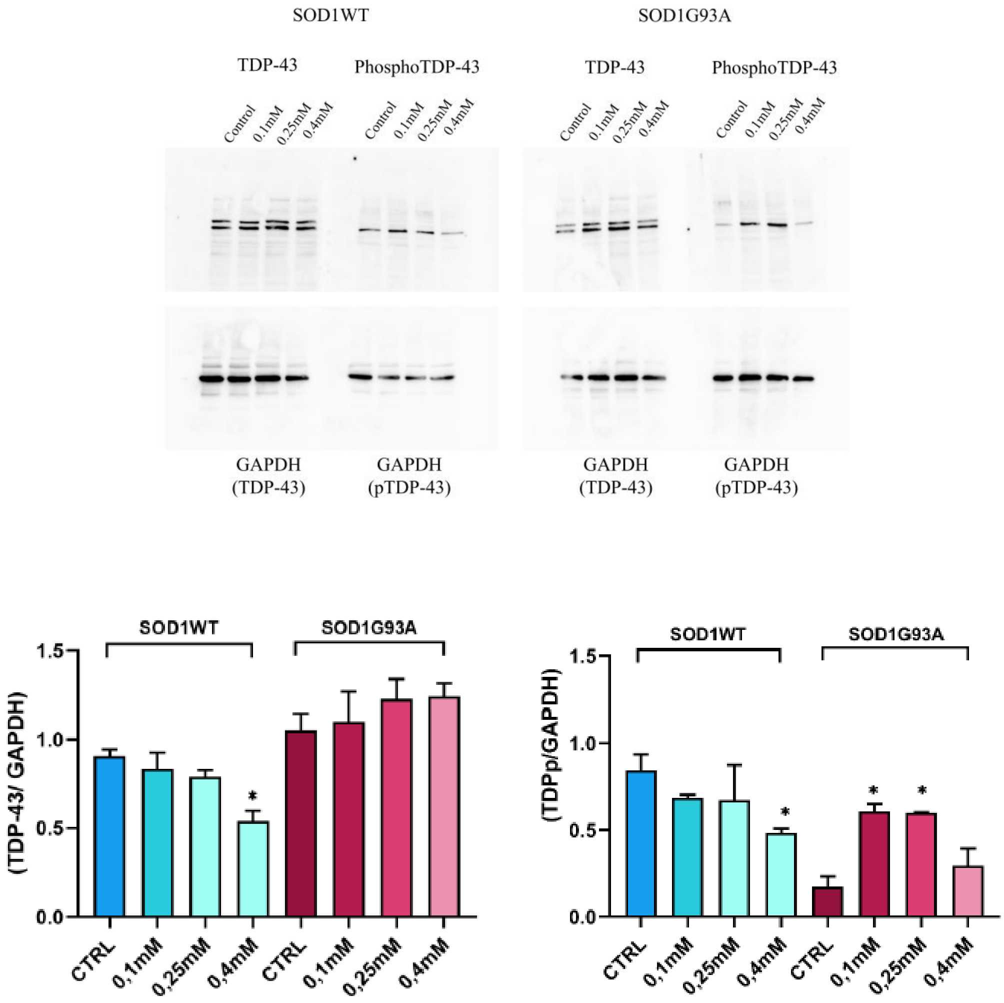

Figure S2: Representative images of immunoblotting membranes for TDP-43 and phospho-TDP43 (TDP-43p) in cells expressing WT or G93A SOD1 under control condition or after exposure to 0.1, 0.25 or 0.4 mM MGO. Total TDP-43 and phosphorylated TDP-43 (TDP-43p) levels were determined and normalized to GAPDH. Statistical analysis was performed using two-way ANOVA followed by Tukey's multiple com-parison test; *p < 0.05 (control X MGO in each strain).

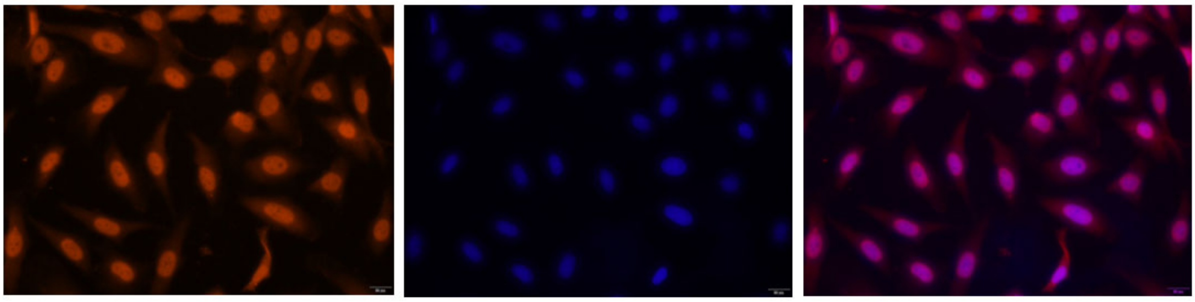

Figure S3: Representative images of H4 cells. Phospho-TDP-43 is shown in red and cell nuclei were stained with DAPI (blue). The last panel shows the merged image of both signals. Phospho-TDP-43 is observed in both the nucleus and the cytosol.
